# Hair Dye Poisoning in a Paediatric Patient

**DOI:** 10.1155/2012/931463

**Published:** 2012-08-16

**Authors:** Jolly Chandran, Rimi Manners, Indira Agarwal, Kala Ebenezer

**Affiliations:** ^1^Paediatric Intensive Care Unit, Department of Paediatrics, Christian Medical College, Vellore 632004, India; ^2^Paediatrics Unit 2, Christian Medical College, Vellore 632004, India

## Abstract

Hair dye ingestion with suicidal intention has increased among rural Indian population and is associated with significant mortality. We report a teenager who presented with cervicofacial edema, respiratory distress, rhabdomyolysis, and myocarditis after ingesting the hair dye Super Vasmol 33. Early and supportive treatment can prevent morbidity and mortality.

## 1. Introduction

Hair dye ingestion as a means of deliberate self-harm is well reported [1–7] and a growing trend is observed among rural Indian population [6–8] but rarely encountered in children.

The ingredient in most hair dyes is paraphenylenediamine (PPD) in concentration ranging from 2 to 10%. The effects of PPD when ingested are serious and are cervicofacial edema, mucosal injury, respiratory distress, acute renal failure, rhabdomyolysis, and myocardial injury [2–7].

Hair dyes are available in stone, powder, or liquid forms. While the liquid forms are more often ingested with suicidal intentions, mortality is higher with the stone forms [7].

We describe the case of a 13-year-old girl who was brought after consumption of hair dye Super Vasmol 33.

## 2. Case

A 13-year-old girl was noticed by her parents lying unconscious home with blackish staining of her fingers and an empty bottle of Super Vasmol 33 (kesh kala: kesh meaning hair, kala meaning black) hair dye by her side. She was taken to a nearby hospital where she developed respiratory distress and was intubated, given gastric lavage, and transferred to our hospital.

On arrival, she was noted to have chemosis and striking facial edema extending to the neck. She was tachycardic with heart rate of 180 beats per minute. Blood pressure was 80/30 mmHg. Third heart sound was audible with a gallop. Continuous bladder drainage was placed which drained cola coloured urine. She was resuscitated with 40 mL/kg of normal saline and was admitted to the Paediatric Intensive Care Unit (PICU).

Her investigations showed a hematocrit of 41.7%. Total white cell count was elevated (2.26 × 10^9^/L) with 92% neutrophils. Platelet count was 211 × 10^9^/L. Serum sodium was 144 mmol/L and potassium was 3.3 mmol/L. Serum creatinine was 1.6 mg/dL. Serum creatinine phosphokinase (CPK) was markedly raised (52834 U/L) with high levels of its MB fraction (CKMB-500 ng/mL); troponin T (cTnT) was also very high (2389 pg/mL). Liver function test showed elevated transaminases, (AST-2529 IU/L, ALT-424 IU/L, normal 8–40). Total bilirubin was 37.6 *μ*mol/L with a direct fraction of 13.6 *μ*mol/L. Total protein and albumin were 79 g/L and 43 g/L, respectively. Serum alkaline phosphatase was normal. Serum calcium was low (6.1 mg%) and phosphate was 3.5 mg%. Venous blood gas analysis showed pH 7.41, pCO2 5.7 kPa, pO2 4.8 kPa, HCO3 26 mmol/L, and BE 2.7 mmol/L. Serum lactate was 2.2 mmol/L and Met Hb was 1 gm%. Urinalysis revealed only RBC 2–4/HPF, but tested positive for haem.

In the intensive care unit she was placed on a ventilator; dopamine and dobutamine infusions were commenced. In view of rhabdomyolysis, the maintenance intravenous fluids were increased to 2 litre/m^2^ and bicarbonate was added. Urine output and pH and CPK levels were monitored. CPK rose to 86940 U/L at 12 hours but hemodialysis was deferred as her urine output remained good. By 36 hours CPK levels dropped to 30082 U/L and serum creatinine improved to 1.2 mg/dL. She gradually improved with supportive care and was extubated on day 4 and later transferred to the ward.

She was discharged from the hospital on the 16th day, at which time she was walking with support and taking liquid diet. ECHO done on day 10 showed normal left ventricle size and function (ejection fraction 57%). At followup she was on normal diet and had no sequelae.

## 3. Discussion

Consumption of hair dye as a deliberate means of self-harm has been reported from different regions in India [1–7], Asia [9], and Africa [10, 11], among adult patients and Sudanese children [11].

Rural, young poor women are the common subjects [6–8, 10] for whom this agent is inexpensive and easily available [3, 6, 7, 10]. The brand Super Vasmol 33 is an emulsion containing 4 g of PPD in 100 mL costing only Rs. 35/-. Hair dyes could be perceived as “not bad enough to kill” by the vulnerable victims who may be taking it just with an intention of threatening the family. Unlike the other commonly used organophosphates, hair dye can be bought without raising suspicion of suicidal intentions, particularly in small villages with closed communities. PPD is thought to have abortive effect in rural Africa [10].

PPD is the permanent black colouring agent applied with ammonia and hydrogen peroxide in hair dying [3, 4, 6, 8]. It is also added to henna (*Lawsonia alba*) and used in the popular tattooing for its darkening effect [4, 10, 11]. PPD is shown to cause rhabdomyolysis in rats by promoting leakage of calcium ions from the smooth endoplasmic reticulum resulting in prolonged muscle contraction and irreversible change in muscle structure [5, 6].

The most marked presentation as has been described of cervicofacial edema was evident in our patient at presentation. The edema of face, neck and laryngeal region could be severe enough to cause respiratory distress, hypoxia, and necessitate an early and emergency intubation or tracheostomy which is life saving in such patients [3–8, 10].

Rhabdomyolysis can also be severe and if associated with high CPK levels can have an adverse outcome [6]. Our patient, however, responded to diuresis and improved without dialysis in spite of high initial CPK levels and serum creatinine. Acute renal failure is a major complication of PPD poisoning [3, 5, 9, 11] and occurs due to a combination of hypovolemia, toxic injury, and myoglobinuria [5, 6]. Renal biopsy studies have shown acute tubular necrosis being the most common finding [1, 3, 5, 7–9] followed by myoglobin casts and interstitial nephritis [1, 3, 7, 9]. Anuria was associated with poor prognosis requiring prolonged hemodialysis in many patients [7].

Myocardial damage and myocarditis are reported less frequently in hair dye poisoning but associated with higher mortality [7, 12]. Very high levels of cTnT and CKMB were suggestive of myocarditis or rhabdomyolysis. Presence of myocardial involvement which until recently was always confirmed only on autopsy is now diagnosed by echocardiography. Myocardial infarction was demonstrated by angiocoronarography in 2006 [13]. Elevated levels of cTnT and CKMB are highly predictive for myocarditis [12, 14].

Resorcinol, Propylene glycol, liquid paraffin, cetostearyl alcohol, sodium lauryl sulfate, ethylenediaminetetraacetic acid (EDTA) sodium, herbal extracts, preservative, and perfume are other ingredients present in small amounts [8] in Super Vasmol. Resorcinol is a corrosive, acid, and protein denaturant causing severe burns on direct contact. Consumption leads to mucosal injury, hypotension, pulmonary edema, methemoglobinemia, hemolysis, metabolic acidosis, convulsion, and liver and renal toxicity [4, 15].

Toxic effects of these ingredients could have had additive effect in our patient. Hair dye (Super Vasmol 33) poisoning is emerging as a suicidal poison that is available quite freely and extensively. There is no specific antidote for PPD and treatment is supportive [2, 4, 5, 8]. Early treatment can prevent renal failure [2, 4, 5]. However, therapeuticdialysis and supportive therapy can result in complete recovery in those who developed renal failure [1, 5, 9]. Paediatricians also have to be aware about the clinical manifestations and emergency airway management of this condition.
